# The Need for Play

**Published:** 1919-04-19

**Authors:** 


### THE NEED FOR PLAY.

Dr. M. Jane Reaney, in a letter to the *Manchester Guardian*, calls for a Director of Play. She says: "We need a Director of Recreation under the Ministry of Health, and in every large town a Supervisor of Recreation, whose duty it will be to ascertain the needs of the children, the young people, and the adults, to know how to satisfy these needs, to organise the facilities so that every one shall have a place to play in. In every town there are sufficient parks, open spaces, playgrounds, and halls to make this possible.

"Physical drill is all very well in its place, but it should be only remedial. If we give the child the chance of free play, of learning and playing the old folk-games and others, and of making use of his natural instinct to dance, if we give the adolescent and the young adult the chance of playing rather than watching the national games, we shall go far to produce a healthy and happy community."

The suggestion has our hearty sympathy and approval. In our review of Sir George Newman's report of the medical work of the Board of Education we drew attention to the awakening of that Board to the importance of games in the scheme of school teaching, and we pointed out how much good had sprung from the organisation of play for the men at rest behind the lines. It is to our county schools that we must look to teach the children how to play. But first our teachers must learn that in any well-ordered scheme of life play has a part as important as that of work. In the past the thinkers and leaders of the nation have laid too much stress upon work. They started our system of national education with the idea only of increasing the capacity of the nation for work. They took no heed of the leisure-time of the people, except to provide opportunities for more book-learning. It was left to Sir Robert Baden-Powell to fill up this gap in our educational scheme, and the Boy Scouts and Girl Guides have learnt more that is worth knowing from him and his Scoutmasters than their schools ever taught them.

Despite our reputation as a sport-loving nation, it is doubtful if the bulk of our people has ever had the time and inclination to play.

We stand to-day at the beginning of great changes in the life of the mass of the people. In the near future there will be a great shortening in the hours of labour of all classes of workers. In the majority of cases the justice of the demand cannot be gainsaid. The nation will gain greatly in health and happiness by its satisfaction if the demand is kept within reasonable limits and if the leisure-time is well spent. Recent investigations into the curve of fatigue have given us the means to determine for each occupation the hours of work which give the greatest production. The invention of labour-saving machinery and its greater use should enable us to cut these hours shorter. Even a loss in production may be faced cheerfully if it provides us with a healthier and happier race. But little good will be gained by the lessening of the hours of work unless the leisure-time of the workers is well spent. We have to teach a nation unused and unapt to play. If Directors and Supervisors of Play are to be of any use to us they must be men and women of wide sympathy and catholic tastes. They must enrol cranks and enthusiasts of all kinds. The man who finds in billiards the highest expression of human effort must work with the man who sees no good in anything except cross-country running, the lover of academic art with the cubist, the card-player with the man who finds his pleasure in working at the lathe, the booklover with the wrestler.

Very closely wrapped up with the teaching of the nation to use its leisure-time wisely and happily is the reform of the public-house. The public-house should be the district club, with eating-rooms and social rooms, card-rooms and billiard-rooms, dancing-hall, library, gymnasium, and playing-fields—a place where men and women could refresh themselves with tea or coffee, beer, whisky, or wine as pleased their palates. In such an atmosphere excess would be frowned upon as anti-social, ugly, and of bad taste. It says much for our innate good sense that drunkenness is not more rife than it is. Our law-makers have made of most of our public-houses places in which there is every temptation to drink to excess. Is it not time they realised that the ordinary Englishman likes a glass of beer and likes to drink it socially and in comfort? Is it not time they learnt that public opinion would crush out tendencies to excess fostered by a system of restriction which brands the public-house as a place of evil and keeps from its doors many who would use it wisely and make it a centre of social life and recreation?

